# Socioeconomic Status, Mental Health, and Workplace Determinants among Working Adults in Hong Kong: A Latent Class Analysis

**DOI:** 10.3390/ijerph18157894

**Published:** 2021-07-26

**Authors:** Alan C. Y. Tong, Emily W. S. Tsoi, Winnie W. S. Mak

**Affiliations:** Department of Psychology, The Chinese University of Hong Kong, Shatin, NT, Hong Kong; alantcy311@gmail.com (A.C.Y.T.); emilytsoi@cuhk.edu.hk (E.W.S.T.)

**Keywords:** latent class analysis, intersectionality, working adults, social factors, well-being, work stress

## Abstract

This study provides insights on mental health correlates and work stress patterns in a representative sample of working adults in Hong Kong using an intersectional perspective. Using data from a cross-sectional, population-based telephone survey of 1007 working adults in Hong Kong, latent class analysis was conducted to identify socioeconomic classes within the sample. Three latent classes were identified, and they differed significantly in all the SES variables. Results suggested mental health to be the lowest in Class 1, the lowest income group. The three classes did not differ from their perceived level of job demand and control in work-related stress. Predictably, the highest income group perceived the lowest level of effort-reward imbalance. The lowest paid class was also reported perceiving the lowest level of relational justice. Different barriers to mental health services were also identified. Finally, cultural implications associated with work stress patterns, research, and practice implications are discussed. This study provides an empirical foundation for future studies to investigate patterns of job stress and mental health needs in a diverse population of working adults, with a particular focus on addressing the intersectional profiles of working adults and their needs in mental health services.

## 1. Introduction

### 1.1. Workplace Mental Health in Hong Kong

A full-time working adult typically spends about 37% of their day at work, and at least 11% of working adults across the globe work as much as 50 h per week [1]. Work is an essential part of an adult’s life, and stressors from work contribute to mental ill-health. Some stressors are associated with “day-to-day” operations, such as job performance, interpersonal conflicts, and systemic pressure [2,3]. Some are concomitant with the work setting, such as mental health stigma, lack of support at work, or workplace bullying [4,5,6,7].

Hong Kong is a high-income city notorious for its work culture with long working hours and high levels of stress [8,9,10,11]. A survey [12] showed that workers in Hong Kong work an average of 50.1 h per week, which is 35% higher than the global average among 71 cities, including Beijing, Tokyo, and Paris. Not surprisingly, long working hours, coupled with other factors, have taken a massive toll on the mental health of working adults. A local epidemiologic study revealed that almost 1 in 7 adults experienced symptoms of depression and/or anxiety [13]. In another survey, up to 25% of working adults felt unmotivated, depressed, or hopeless, and a staggering 90% of respondents urged for additional mental health resources at work [14].

### 1.2. Socioeconomic Status and Mental Health

Mental ill-health in the population is a complex issue that is both a cause and result of socioeconomic-related factors, including poverty, limited education opportunities, limited access to health care, and discrimination, to name a few [15]. Socioeconomic status (SES) is a broad term encompassing a spectrum of constructs, including income, industry, housing situation, and educational attainment [16], representing how a society is structured and providing information about an individual’s access to social and economic resources. Notwithstanding the constant debate on the conceptualization and measurement of SES, it is undoubtedly one of the most important explanatory variables for social epidemiologists to address issues concerning mental health or physical health inequities [17]. According to a meta-analysis, lower SES was associated with higher odds for depression [18]. In addition to SES, female gender [19], low educational attainment [20,21], and low income [22] are also consistently associated with increased risks of depression and other mental disorders.

### 1.3. Intersectionality

Although there is evidence illustrating the associations between various socioeconomic and demographic factors of mental health and that membership in one social category is likely to interact with another [23], traditional subgroup analysis often undermines the complexities that cut across sociodemographic categories [24]. Moreover, social inequalities in mental health are suggested to include many interrelated social variables, which, when analyzed in isolation, may lead to “false” associations [25]. In order to ameliorate these methodological shortcomings, an intersectional analysis may offer a new perspective by accounting for concurrent multiple identities.

Intersectionality is a theory that underscores the complexities of different social identities. It holds that a person can be identified with multiple social positions concurrently, and how each position converges or diverges with one another reflects the unique interlocking patterns of oppression and/or opportunities that impact ones’ health and well-being [26]. It challenges us to not just perceive people by any single demographic factor (such as gender or income) but to consider the interaction between coexisting social identities. Intersectionality theory asserts that individuals who are affiliated with multiple disadvantaged groups, such as a transgender older adult who is also an immigrant and living in poverty, are more prone to experience mental ill-health compared with those who belong to only one single disadvantaged group [27].

Studying the influence of SES on mental health with an intersectional perspective is appropriate because every individual is embedded in a multi-leveled ecological system, and their health is largely influenced by their positions in society [28,29]. Therefore, such investigation ought to be intersectional.

### 1.4. Theoretical Framework of Work Stress

Work stress contributes to mental ill-health. Two models of work stress were employed in our study. The job demand-control-support (DCS) model [30] postulates that job strain results from excess demand in comparison with control at work, with social support playing a buffering role in moderating the relationship between job demand and control. In the context of low social support and a high job demand over job control, isostrain can result.

Alternatively, the effort-reward imbalance (ERI) [31] model conceptualizes work stress as resulting from a perceived lack of reciprocity in social exchange, that is, high effort (i.e., skills and energy invested into the job) coupled with low reward (i.e., in terms of income, prestige, recognition, or personal satisfaction). Whereas the ERI model centers on the violation of adequate exchange (monetary or social), the DCS model emphasizes the intrinsic exertion of an individual for his or her job [32]. Therefore, the concurrent use of both models provides a broad picture of work stress.

In addition, poor relationships with supervisors constitute another major source of stress [33]. Therefore, relational justice and the extent of fairness exhibited by a manager were considered in the present study. In the context of workplace well-being, having a high effort-reward and isostrain ratio but perceiving poor relational justice are all negatively linked with work-health outcomes, including work engagement, motivation, and psychological distress [34,35,36]. Past investigations have shown that lower SES status [37,38] and being female [39] were associated with higher levels of work stress. Marginalized social groups, including older adults [40], ethnic minorities [41,42], and sexual minorities [43], also experienced elevated work stress, mostly due to workplace discrimination.

In sum, although evidence suggests that people from different SES experience work stress differently, little is known about how multiple social categories may intersect to manifest different patterns of mental health outcomes and stress among working adults. Specifically, in this study, we focused on six socioeconomic and demographic factors, including age, gender, education, income, job position, and industry. These factors were chosen because previous research, which studied them in isolation, pointed to a need for a more coherent understanding across intersectional profiles [44,45]. This study seeks to provide insights on mental health correlates and patterns of work stress among a representative sample of working adults in Hong Kong from an intersectional perspective.

## 2. Materials and Methods

### 2.1. Study Design and Participants

As a part of a large-scale community-based initiative promoting mental health among working adults in Hong Kong, this study is a cross-sectional, territory-wide, population-based survey. Participants were recruited from a random sampling of both landline and mobile numbers. The survey targeted Cantonese-speaking residents in Hong Kong who were 18-years-old and above who have been working full-time in the past 12 months at the time of the survey. The survey was administrated by the Hong Kong Public Opinion Research Institute (PORI), an institution independent of the research team that specialized in conducting telephone surveys. Ethics approval was obtained from the institutional review board at the author’s institution. (Ref: SBRE 19-129).

### 2.2. Data Collection

A total of 29,898 call attempts were made between February and March 2020. Landline and mobile telephone numbers were randomly generated. After checking for eligibility of successful call attempts, 1007 valid responses were received (response rate: 57.2%) and retained for analysis. A valid respondent was defined as someone who answered at least 70% of the questions. The characteristics of these respondents were included in the “entire sample” column in tables from the Supplementary Materials.

### 2.3. Measurements

During the phone interviews, demographic information was obtained, including the highest level of education attained, monthly income in Hong Kong dollars (HKD), job position, industry, age, and gender. Total working hours per week and experience of workplace bullying were also asked. In addition, respondents were assessed on a battery of mental health indicators, including their depressive and anxiety symptoms, flourishing, help-seeking intentions, and behaviors. Moreover, respondents were invited to share their views on workplace mental health resources. Lastly, work factors including relational justice, job-demand-control, and effort-reward imbalance were assessed. Appendix A details the format and scoring of these measurements.

### 2.4. Statistical Analysis

A latent class analysis (LCA) was performed using Mplus version 7.0 [46] to identify SES classes within the sample. LCA is a person-centered approach congruent with the ideology of intersectionality. It detects homogeneous subpopulations of individuals that are latent via the intersection of different characteristics [47]. Models were evaluated based on conceptual meaning and the following fit indices: the Akaike information criterion (AIC; [48]), Bayesian information criterion (BIC; [49]), and the sample size adjusted Bayesian information criterion (SSABIC; [50]). The Vuong–Lo–Mendell–Rubin likelihood ratio test (VLMR LR; [51]) and the Lo–Mendell–Rubin adjusted likelihood ratio test (LMR LR; [52]) were used to compare models. Since the exact number of latent classes representing SES was unknown, an exploratory approach was employed starting with the single-class model and fitted successive models with an increasing number of classes until the LMR was no longer significant. Missing data were handled by maximum likelihood estimation. After the number and nature of classes were determined, the differences in mental health outcomes as well as work-related variables between classes were compared using one-way ANOVA (for continuous variables) and Chi-square tests (for categorical variables) in SPSS version 26.0. Significant differences were followed by post hoc pairwise comparisons with Bonferroni’s adjustment.

## 3. Results

### 3.1. Class Enumeration

LCA models with classes ranging from 1 to 4 were fitted based on six socioeconomic and demographic factors. The model summary is listed in Table 1. Results revealed that a 3-class model was most theoretically meaningful and acceptable, accounting for the indication of various fit indices. The first nonsignificant LMR LR and ALMR LR were obtained in this solution. Although the AIC, BIC, and SSABIC were not the smallest, the values were smaller compared with the 1-class and 2-class models. Altogether, the fit indices indicated that the 3-class model was preferable to other models. Table 2 shows the item-response probability for this model.

### 3.2. Class Characteristics

Results showed that the three classes differed significantly on all of the socioeconomic and demographic variables. Detailed statistics are shown in Table 3.

In terms of gender, Classes 1 and 2 had more male respondents, whereas Class 3 had significantly more female respondents. In terms of education, individuals in Class 2 and 3 were highly educated, as more than 90% received tertiary education, whereas approximately half of the individuals in Class 1 had. As for age, people in Class 1 and 3 were older and Class 1 had the highest percentage of people in their 50s. People in Class 3 were the youngest, with more than 90% aged under 40 years old.

The three classes also differed significantly on monthly income, job position, and industry. Over 80% of people in Class 3 and about half in Class 1 earned a mid-range monthly income (HKD 15,000–39,999; or USD 1875–4999). Most people in Class 2 had relatively higher income of HKD 40,000–69,999 (or USD 5000–8750) and were higher in their job position as professionals, managers, or executives. Note that although people in Class 3 earned less compared with those in Class 2, a great proportion of them (41%) also held high positions in the hierarchy, such as being entrepreneurs, managers, and professionals. In terms of industry, more people in Class 1 worked in the construction/manufacturing industry. More people in Class 2 and 3 worked in the commercial or professional industries.

The classes were named based on their distinct socioeconomic features collectively, as mentioned above. Specifically, Class 1 was named *Laborers* since people in this class predominately engage in moderately valued paid work that requires physical strength rather than professional titles; Class 2 was referred to as *Established leaders* since most people in this class are middle-aged and hold highly skilled or management positions; Class 3 was named *Emerging executives* as people in this class are highly educated, young, and also hold high positions and executive roles.

### 3.3. Mental Health

Table 4 shows detailed statistics of respondent’s mental health-related indicators. Depression levels significantly differed across the three classes. Follow-up test results revealed that only the difference between established leaders and emerging executives was significant. Emerging executives had significantly higher levels of depressive symptoms (M = 1.16, SD = 1.39) than established leaders (M = 0.85, SD = 1.25), as assessed by PHQ-2 total score. With three points as the cut-off, *laborers* had the highest percentage of people meeting the cut-off (N = 67, 17.1%) for probable clinically significant depressive symptoms. Flourishing levels also differed across classes. Established leaders (M = 5.64, SD = 0.88) had the highest flourishing scores and *laborers* (M = 5.25, SD = 0.94) had the lowest. The difference between *laborers* and *emerging executives* was not significant in the post hoc test. As assessed by GAD-2 total score, anxiety levels did not differ significantly across classes when the scores are treated as continuous variables or binary variables with the clinical off score of three. Both help-seeking intention and behaviors differed significantly across the classes. *Laborers* had the lowest help-seeking intention (M = 5.47, SD = 2.35) and behaviors (M = 2.80, SD = 2.78) among all.

### 3.4. Work Variables

Table 4 also shows detailed statistics of respondent’s work-related indicators. Both *established leaders* (M = 45.82, SD = 10.61) and *emerging executives* (M = 45.75, SD = 10.23) work for about 46 h each week. *Laborers* work significantly longer hours (M = 48.73, SD = 13.53). In terms of workplace bullying, *established leaders* reported the most workplace bullying experience either previously or currently, and significantly more than *emerging executives*. Only 55.2% of *established leaders* reported having never been bullied, compared with *emerging executives* (64.9%) and *laborers* (69.5%).

Regarding work stress, *established leaders* had the least effort-reward imbalance ratio (M = 1.19, SD = 0.48) compared with others. Significantly lower levels of relational justice (M = 3.49, SD = 0.92) was perceived among the *laborers*. Job strain did not significantly differ across classes.

### 3.5. Appraisal of Mental Health Resources

Table 5 presents responses about workplace mental health resources. Less than one-third of the respondents (*n* = 291, 28.9%) agreed that sufficient mental health resources were provided at work. About 36% of them (*n* = 366) mentioned their employers did not provide any resources on mental healthcare at all, while 21.6% (*n* = 218) acknowledged some resources available but they felt insufficient. *Laborers* had the highest proportion of people expressing that they were not supported (41%). More *emerging executives* rated having insufficient mental health resources (29.4%) compared with the others. In terms of usage, only 12.6% (*n* = 127) of the entire sample were using the mental health resources provided at the time of interview. Nevertheless, most others (*n* = 728, 72.3%) expressed willingness to use when in need.

About 13% (*n* = 139) were non-users who refused to use any mental health resources provided at work even when in need. Their reasons for not using such resources were followed up by an open-ended question. Their responses were organized into six identifiable themes to facilitate interpretation. Most non-users (60.9%) expressed no need of mental health resources. About 25% refrained from using due to a lack of trust in related services. Accessibility barriers and fear of disclosure to different parties contributed a similar proportion of 11–12%. Other reasons included a lack of mental health literacy and fear of potentially negative consequences for their career. It was noteworthy that *emerging executives* had significantly fewer non-users (10.7%) than the others, and far fewer of these executives expressed a lack of need for mental health services. Yet, a higher proportion of them expressed having a lack of trust toward mental health services.

In terms of preferred resources, most people welcomed mental health-related conditions to be covered by medical insurance (*n* = 651, 65%) and corporate policies catering to mental ill-health conditions (*n* = 635, 63%). Similar proportions of people preferred seminars/workshops (*n* = 413, 41%) and in-house psychologists/coaches (*n* = 386, 38%) as means of support. About a quarter (*n* = 281, 28%) wanted online courses. Other preferences represented only a small fraction (<1%) of the total responses.

## 4. Discussion

This study examines mental health and work stress patterns in a population-based sample of working adults in Hong Kong. Three latent classes were identified: (1) a relatively low-income group with the longest working hours (*laborers*); (2) a group of adults mostly with managerial positions in their respective industries (*established leaders*), and (3) a group of younger adults (over 90% millennials) who are highly educated, hold high job positions but are less financially established than their older counterparts (*emerging executives*). Results revealed distinct patterns of mental health outcomes in these three groups of individuals. Overall, our findings showed that those from the lowest SES group (*laborers*) had the worst mental health.

We used three indicators to infer the levels of job stress. Significant differences were found in both effort-reward imbalance and perceived relational justice across the three groups, but not in job strain. Job strain and effort-reward imbalance may be seemingly interrelated constructs, but differential results suggested they may be independent of each other [53]. Our findings suggested that these working adults shared similar demand-control experiences at work. Typically, workers in Hong Kong were found to not participate in decision making [54], possibly under the influence of traditional values that emphasize the virtues of submission, humility, tolerance, and hierarchy [55]. With top-down decision making and tight schedules being more likely to be tolerated, the perceived significance of demand-control diminishes. At the same time, Hong Kong workers were also found to weigh their pay heavily on top of other things, such as interests and learning opportunities [56,57]. When income became salient in evaluating effort-reward balance, it was not surprising that *established leaders* viewed themselves as more balanced with their higher earnings.

*Laborers* reported the lowest levels of perceived relational justice at work among the workers, suggesting that their supervisors might not have exhibited sufficient openness and respect; this is probably because fewer *laborers* worked in the business and professional sectors where leadership competency has been advocated for a long time. Leaders in the business and professional sectors may have received relevant training and thus experienced a comparatively higher level of perceived relational justice among *established leaders* and *emerging executives*.

The association between SES and bullying may be revisited when intersecting with work roles. The *established leaders* in this study reported the highest prevalence of previous and current workplace bullying, in contrast with past evidence affirming that employees from the lower SES strata, who work in precarious conditions, were more vulnerable to workplace bullying [58,59]. This finding was unexpected but is considerable with the following in mind. First, managers face fierce competition over the course of their career to rise to a higher position within the organizational hierarchy, and competition is often associated with some degree of aggression and bullying. Second, since Asian employees, in general, are more likely to conform rather than voice their dissatisfaction [54], managers may feel caught in the middle as they are still somewhat limited in their autonomy and must gain support from both their supervisors and staff. A study from Poland [60] found that individuals with managerial jobs experienced bullying more often than those with non-management positions, echoing our findings.

All in all, our results appeared to confirm previous findings that lower SES statuses were associated with higher work stress and can link negatively with work-health outcomes, including mental health outcomes [36]. Findings on workplace bullying were unexpected yet understandable and may warrant further investigation to uncover different correlates.

### 4.1. Limitations

This study has several limitations. For instance, this study did not purposefully sample people from minority groups, such as ethnic and sexual minorities, whose membership in those groups may cause them to have different workplace experiences. Future studies should include ethnicity, sexual orientation, and gender identity as other intersecting identities that may affect workplace mental health.

Additionally, we used multiple objective indicators to measure the SES of our participants. Although the conceptualization and measurement of SES have always been controversial among social epidemiologists, some researchers urge the inclusion of both objective and subjective SES measures, as subjective SES was shown to be a significant mediator between objective SES and subjective well-being [61]. Depending on the outcome, variables of interest and future research may include a broad range of both objective and subjective SES indicators to improve the specificity of their results.

### 4.2. Research and Practical Implications

Even though the high variability in our sample’s occupational characteristics causes some difficulty in summarizing the results, our findings showed pressing mental health needs across classes. The salient question that future research could explore is: What are some of the ways in which mental health services can strategically cater to the needs of working adults?

Our study offers several pointers for mental health service providers and policymakers. Out of the mental health support suggestions provided, the type of supports preferred did not differ across the three groups. Specifically, 65% of respondents preferred, first and foremost, to receive healthcare insurance coverage for mental health-related costs, followed by mental health-friendly policies in the workplace (63%). Health insurance that can sufficiently cover mental health care costs remains a gap to be filled. In Hong Kong, an average appointment with a private clinical psychologist costs about HKD 1000–2000 per hour (equivalent to USD 125–250), and the fee is even higher for private psychiatrists (HKD 1000–4000 per consultation; or USD 125–500). An average working adult may find these services unaffordable and resort to cheaper outpatient public psychiatric services (HKD 80; or USD 10 per visit). However, public psychiatric services are notorious for their lengthy waiting time of months or even surpassing one year, depending on urgency. Currently, most major insurance companies in Hong Kong (e.g., Bupa, AIA, BlueCross) have launched health protection plans with coverage for mental health treatment-related costs. Unfortunately, there is no available data to show the extent of corporations purchasing these plans for their employees and their level of acceptability of paying for such plans.

Our study identified two major barriers to service: fear of disclosure and a lack of trust in mental health services. Under the new normal of working remotely and maintaining personal relationships via the Internet, blended care or e-mental health may be considered an imminent and robust solution to the said concerns. E-mental health enables users to seek out services anonymously, encourage self-management and treatments to be accessed at their own time and pace in a cost-effective manner [62]. Recent reviews have revealed the efficacy of e-mental health services in alleviating depressive and anxiety symptoms as well as its effectiveness in enhancing well-being for working adults [62,63]. In addition, tech-savvy millennials (born between 1980–2000s) will comprise approximately 50% of the global workforce by year 2025. Considering that the youngest group, *emerging executives*, expressed a greater need for services but felt a sense of distrust, more research effort should therefore be directed into understanding how mental health services can be tailored to millennials who may have differences in preferences and utility patterns than their older counterparts.

## 5. Conclusions

This research provided insights for researchers interested in understanding the intersectionality of work-related stress and SES and demographic profiles in a sample of working adults in Hong Kong. Three classes of working adults were identified and each presented distinctive characteristics. Specifically, the *laborers* worked in a relatively more oppressive environment, as reflected in their long working hours, high effort-reward imbalance, and poor relational justice. They have poor mental health and yet are less willing to seek help. The *established leaders* flourished the most but also reported the highest level of bullying experience. *Emerging executives* are young adults who hold high positions but are less mentally healthy. They have an ambivalent attitude toward mental health services and support at work. Broadly consistent with intersectionality theory, our results confirm that patterns of work-related stressors and mental health outcomes are affected differentially among intersecting social identities. For example, younger participants (but not older) with higher positions are more ambivalent than their older counterparts in their attitudes toward mental health services, and older participants with higher positions are more mentally healthy, but not vice versa. Notwithstanding its limitations, this study provides an empirical foundation for future studies to investigate patterns of work stress and mental health needs in the diverse population of working adults, with a particular focus on addressing their intersectional profiles and needs in mental health services.

## Figures and Tables

**Table 1:** Comparisons of LCA models with different number of latent classes.

Model	Log-Likelihood	AIC	BIC	SSABIC	Entropy	Class Count of the Smallest Class	LMR LR *p*-Value	ALMR LR *p*-Value	BLRT *p*-Value
1-Class	−7111.81	14,263.62	14,361.91	14,298.39	/	1007	/	/	/
2-Class	−6811.29	13,704.57	13,906.08	13,775.86	0.675	488	0.0019	0.002	<0.0001
**3-Class**	**−6645.91**	**13,415.81**	**13,720.53**	**13,523.61**	**0.801**	**283**	**0.0206**	**0.0211**	**<0.0001**
4-Class	−6539.02	13,244.04	13,651.96	13,388.35	0.816	140	0.839	0.8401	<0.0001

Note. AIC: Akaike information criterion; BIC: Bayesian information criterion; SSABIC: sample-size adjusted Bayesian information criterion; LMR LR: Lo–Mendell–Rubin likelihood ratio test; ALMR LR: Adjusted Lo–Mendell–Rubin likelihood ratio test; BLRT: bootstrap likelihood ratio test (100 bootstrap draws).

**Table 2:** Item response probability for a 3-class model.

Variable	Scale/Category	Laborers (*n* = 392)	Established Leaders (*n* = 332)	Emerging Executives (*n* = 283)
Latent class prevalence	-	0.38	0.34	0.28
Gender	Male	**0.54**	**0.58**	0.42
	Female	0.47	0.42	**0.58**
Income (HKD)	$14,999 or below	0.30	<0.01	0.12
	$15,000–$39,999	**0.66**	0.25	**0.83**
	$40,000–$69,999	0.03	0.46	0.05
	$70,000 or above	0.01	0.28	<0.01
Highest education attainment	Below primary	0.06	<0.001	<0.001
	Secondary	**0.90**	0.11	<0.01
	Tertiary	0.03	**0.89**	**0.99**
Age	18–29	0.10	0.02	**0.59**
	30–39	0.19	0.25	0.30
	40–49	0.25	0.38	0.10
	50–59	0.32	0.29	0.02
	>60	0.14	0.06	<0.01
Position	Professional, Managers, Executive	0.18	**0.82**	0.43
	Self-employed/Entrepreneurs	0.05	0.08	0.01
	Office/Non-office skilled	0.36	0.07	0.30
	Office/Non-office Non-skilled	0.41	0.03	0.25
Industry	Commercial Sector	0.14	0.31	0.21
	Semiprofessional/Professional	0.12	0.38	0.32
	Hospitality	0.15	0.03	0.04
	Retail and Sales	0.17	0.07	0.09
	Construction/Manufacturing	0.20	0.14	0.11
	Public Services	0.05	0.06	0.09
	Media	0.02	<0.001	0.08
	Logistics/Transport	0.14	0.02	0.06

Note. Item response probability > 0.50.

**Table 3:** General Demographics of Respondents.

		Entire Sample (*n* = 1007)	Laborers (*n* = 392)	Established Leaders (*n* = 332)	Emerging Executives (*n* = 283)	Between Class Differences	Post Hoc Tests/Pairwise Comparisons ^1^		
Variable	Scale/Category	n(%)/M(SD)	n(%)/M(SD)	n(%)/M(SD)	n(%)/M(SD)	$\chi^{2}$	L vs. EL Mean Diff/ $\chi^{2}$	L vs. EE Mean Diff/ $\chi^{2}$	EL vs. EE Mean Diff/ $\chi^{2}$
Gender	Male	524 (52)	210 (53.6)	200 (60.2)	114 (40.3)	24.99 ***	3.26	11.63 **	24.35 ***
	Female	483 (48)	182 (46.4)	132 (39.8)	169 (59.7)				
Income (HKD)	$14,999 or below	140 (13.9)	107 (29.7)	0 (0)	33 (12.2)	616.00 ***	412.31 ***	29.92 ***	324.15 ***
	$15,000–$39,999	532 (52.8)	242 (67.2)	65 (21.6)	225 (83.3)				
	$40,000–$69,999	169 (16.8)	9 (2.5)	148 (49.2)	12 (4.4)				
	$70,000 or above	90 (8.9)	2 (0.6)	88 (29.2)	0 (0)				
Highest education attainment	Below primary	24 (2.4)	24 (6.2)	0 (0)	0 (0)	873.71 ***	594.18 ***	645.88 ***	24.05 ***
	Secondary	386 (38.3)	359 (92.3)	27 (8.2)	0 (0)				
	Tertiary	590 (58.6)	6 (1.5)	303 (91.8)	281 (100)				
Age	18–29	206 (20.5)	36 (9.3)	3 (0.9)	167 (59.4)	485.40 ***	47.83 ***	291.41 ***	341.50 ***
	30–39	236 (23.4)	73 (18.9)	76 (23)	87 (31)				
	40–49	250 (24.8)	96 (24.8)	131 (39.7)	23 (8.2)				
	50–59	229 (22.7)	127 (32.8)	99 (30)	3 (1.1)				
	>60	77 (7.6)	55 (14.2)	21 (6.4)	1 (0.4)				
Position	Professional, Managers, Executive	465 (46.2)	77 (19.9)	273 (82.7)	115 (41.1)	357.61 ***	338.58 ***	40.34 ***	184.58 ***
	Self-employed/Entrepreneurs	50 (5)	16 (4.1)	31 (9.4)	3 (1.1)				
	Office/Non-office skilled	244 (24.2)	138 (35.7)	19 (5.8)	87 (31.1)				
	Office/Non-office Non-skilled	234 (23.2)	153 (39.5)	7 (2.1)	74 (26.4)				
Industry	Commercial Sector	210 (20.9)	55 (14.4)	103 (31.7)	52 (18.9)	202.93 ***	142.45 ***	93.66 ***	48.07 ***
	Semiprofessional/Professional	258 (25.6)	49 (12.8)	116 (35.7)	93 (33.8)				
	Hospitality	77 (7.6)	56 (14.6)	10 (3.1)	11 (4)				
	Retail and Sales	113 (11.2)	64 (16.7)	24 (7.4)	25 (9.1)				
	Construction/Manufacturing	151 (15)	77 (20.1)	46 (14.2)	28 (10.2)				
	Public Services	61 (6.1)	18 (4.7)	18 (5.5)	25 (9.1)				
	Media	29 (2.9)	8 (2.1)	0 (0)	21 (7.6)				
	Logistics/Transport	78 (7.7)	54 (14.1)	6 (1.8)	18 (6.5)				
	Others	6 (0.6)	2 (0.5)	2 (0.6)	2 (0.7)				

Note. ** *p* < *0*.01, *** *p* < *0*.001; ^1^ L: Laborers, EL: Established leaders, EE: Emerging Executive.

**Table 4:** Mental health and work-related indicators of respondents.

		Entire Sample (*n* = 1007)	Laborers (*n* = 392)	Established Leaders (*n* = 332)	Emerging Executives (*n* = 283)	Between Class Differences	Post Hoc Tests/Pairwise Comparisons ^1^		
Variable	Scale/Category	n(%)/M(SD)	n(%)/M(SD)	n(%)/M(SD)	n(%)/M(SD)	*F/* $\chi^{2}$	L vs. EL Mean Diff/$\chi^{2}$	L vs. EE Mean Diff/$\chi^{2}$	EL vs. EE Mean Diff/$\chi^{2}$
Mental health indicators									
Depression	PHQ-2 total score	1.04 (1.37)	1.10(1.43)	0.85(1.25)	1.16(1.39)	4.81 **	0.25 *	−0.06	−0.31 *
	PHQ-2 score ≥ 3	145 (14.4)	67 (17.1)	35 (10.5)	43 (15.2)	6.46 *	3.37 *	0.43	2.99
Anxiety	GAD-2 total score	1.39 (1.55)	1.43(1.67)	1.26(1.45)	1.49(1.48)	1.95	/	/	/
	GAD-2 score ≥ 3	187 (18.6)	87 (22.2)	54 (16.3)	46 (16.3)	5.57	/	/	/
Flourishing	FS	5.41 (0.93)	5.25(0.94)	5.64(0.88)	5.38(0.94)	16.02 ***	−0.38 ***	−0.12	0.26 **
Help-seeking	Help-seeking intention	6.06 (2.16)	5.74(2.35)	6.36(1.98)	6.15(2.04)	7.77 ***	−0.62 ***	−0.41	0.21
	Help-seeking behaviors	3.47 (2.92)	2.80(2.78)	4.08(2.95)	3.68(2.90)	18.78 ***	−1.28 ***	−0.88 ***	0.40
Work-related indicators									
Working hours	Hours of work per week	46.93 (11.81)	48.73 (13.53)	45.82 (10.61)	45.75 (10.23)	6.69 **	2.91 **	2.99 **	0.07
Workplace bullying	Yes currently at this workplace	103 (10.2)	44 (11.3)	40 (12.1)	19 (6.7)	16.92 **	7.96	4.22	13.99 **
	Yes previously at this workplace	76 (7.5)	27 (6.9)	31 (9.4)	18 (6.4)	/	/	/	/
	Yes previously at previous workplace	192 (19.1)	66 (16.9)	77 (23.3)	49 (17.4)	/	/	/	/
	Never	631 (62.7)	253 (64.9)	182 (55.2)	196 (69.5)	/	/	/	/
Effort-reward imbalance	ERI	1.30 (0.63)	1.36(0.70)	1.19(0.48)	1.36(0.65)	8.42 ***	0.17 **	−0.01	−0.18 **
Relational justice	RJ	3.61 (0.89)	3.49(0.92)	3.68(0.85)	3.71(0.86)	6.57 **	−0.19 *	−0.23 **	−0.04
Job-demand-control	Iso-Strain	0.26 (0.24)	0.27(0.30)	0.25(0.24)	0.25(0.24)	0.81	0.02	0.02	0.002

Note: * < 0.05, ** *p* < 0.01, *** *p* < 0.001; ^1^ L: Laborers, EL: Established leaders, EE: Emerging executives.

**Table 5:** Appraisal of mental health resources at work.

		Entire Sample (*n* = 1007)	Laborers (*n* = 392)	Established Leaders (*n* = 332)	Emerging Executives (*n* = 283)
Domain	Category	n(%)/M(SD)	n(%)/M(SD)	n(%)/M(SD)	n(%)/M(SD)
Evaluation of resources at work					
Sufficiency of mental health resources provided at workplace	Mean scores	1.32 (1.25)	1.26 (1.27)	1.46 (1.28)	1.24 (1.19)
	No service at all	366 (36.3)	155 (41.6)	110 (34.4)	101 (36.2)
	Insufficient	218 (21.6)	72 (19.3)	64 (20.0)	82 (29.4)
	Neither sufficient nor insufficient	97 (9.6)	39 (10.5)	34 (10.6)	24 (8.6)
	Sufficient	291 (28.9)	107 (28.7)	112 (35)	72 (25.8)
Usage of mental health resources provided at workplace	Will use and is currently using	127 (12.6)	55 (14.3)	38 (11.7)	34 (12.1)
	Will use in the future if needed	728 (72.3)	274 (71.4)	238 (73.5)	216 (77.1)
	Will not use	133 (13.2)	55 (14.3)	48 (14.8)	30 (10.7)
Reasons of not using ^1^					
	No demand for extra support	81 (60.9)	42 (76.4)	31 (64.6)	8 (26.7)
	Lack of trust in mental health services	33 (24.8)	7 (12.7)	14 (29.2)	12 (40)
	Accessibility issue	14 (10.5)	7 (12.7)	1 (2.1)	6 (20)
	Fear of disclosure	16 (12.0)	1 (1.8)	6 (12.5)	6 (20)
	Work-related concerns	3 (2.3)	1 (1.8)	1 (2.1)	2 (6.7)
	Lacking mental health literacy	2 (1.5)	1 (1.8)	0 (0)	1 (3.3)
	Other	4 (3.01)	1 (1.8)	2 (4.2)	1 (3.3)
Needs ^2^					
No such need	N/A	52 (5)	34 (8.7)	13 (3.9)	5 (1.8)
Learning resources	Seminars or workshops	413 (41)	147 (37.5)	148 (44.6)	118 (41.7)
	Online courses	281 (28)	104 (26.5)	93 (28.0)	84 (29.7)
	Continuing education program	1 (0.1)	1 (0.3)	0 (0)	0 (0)
Allowance/financial resources	Medical insurance coverage on mental health conditions	651 (65)	226 (57.7)	214 (64.5)	211 (74.6)
	Fringe benefits	3 (0.3)	1 (0)	1 (0.3)	1 (0.4)
	Salary adjustment	2 (0.2)	1 (0.3)	1 (0.3)	0 (0)
Mental-health friendly policies	Policy catering mental ill-health conditions	635 (63)	241 (61.5)	208 (62.7)	186 (65.7)
	On-site coach/psychologist	386 (38)	120 (30.6)	132 (39.8)	134 (47.3)
	Work-life balance policy	2 (0.2)	0 (0)	1 (0.3)	1 (0.4)

Note. ^1^ n divided by the number of people who answered ‘Will not use’; ^2^ n divided by the total number of respondents.

## Data Availability

Data is available upon written request to the corresponding author.

## Supplementary Materials

The following are available online at https://www.mdpi.com/article/10.3390/ijerph18157894/s1, Tables S1–S5.

## Appendix A

**Table A1:** Measurement Tools.

Construct	Scale	Scoring	Description	Reliability
Mental health-related constructs				
Depressive symptoms	The Patient Health Questionnaire-2 (PHQ-2; Kroenke et al., 2003)	0–3 (0 = “not at all”, 1 = “several days”, 2 = “more than half the days”, 3 = “nearly every day”)	The PHQ-2 was developed based on the long-form as a tool for preliminary screening of depression. The respondents were asked to rate the frequency of occurrence of two depressive symptoms (anhedonia and depressed mood) over the past two weeks by choosing one of the four response options: “0-Not at all”, “1-Several days”, “2-More than half the days”, or “3-Nearly every day”. The total scores range from 0 to 6, and higher scores indicate more severe depressive symptomatology. Using a cut-off of 3, the PHQ-2 has a sensitivity of 82.9% and specificity of 90% for the diagnosis of major depressive disorder.	0.60
Anxiety symptoms	Generalized Anxiety Disorder 2-item (GAD-2; Kroenke et al., 2007)	0–3 (0 = “not at all”, 1 = “several days”, 2 = “more than half the days”, 3 = “nearly every day”)	The GAD-2 is a simple initial screening tool for generalized anxiety disorder developed based on the long-from. It reflects how often the subjects have suffered from the first two core symptoms of generalized anxiety disorder (feeling nervous, anxious, or on edge and unable to stop or control worrying) over the past two weeks. GAD-7 scores range from 0 to 6, with higher scores representing more severe anxiety symptoms. Using a cut-off of 3, the GAD-2 has a sensitivity of 86% and specificity of 83% for diagnosis of generalized anxiety disorder.	0.72
Flourishing	The Flourishing Scale (FS; Diener et al., 2010)	1–7 (1 = “strongly disagree”, 7 = “strongly agree”)	The FS consisted of eight statements measuring the respondent’s self-perceived attainment in important areas such as relationships, self-esteem, purpose, and optimism. The scale provides a single psychological well-being score. Respondents rated the extent to which they agreed or disagreed with the 8 statements relating to their well-being, for instance, “I lead a purposeful and meaningful life”, “My social relationships are supportive and rewarding”, and “I am optimistic about my future”. The higher scores represent a person with many psychological resources and strengths and thus more flourished.	0.82
Help-seeking	Self-constructed	Intention: 0–10 (0 = ‘‘no at all willing’’, 10 = ‘‘most willing’’); Behavior: 0–10 (0 = ‘‘did not attend at all’’, 10 = ‘‘most often’’)	Respondents’ intention toward seeking help was assessed using three self-constructed questions asking whether they were willing to: 1) seek professional help when facing psychological distress; 2) encourage acquaintances to seek psychological services when needed; 3) discuss mental health issues with others. They rated their level of willingness. There was an additional question assessing their actual help-seeking behaviors by asking how often they attend mental health-related activities.	0.73
Workplace mental health resources	Self-constructed	Availability: 1–5, (1 = ‘none’ to 5 = ‘adequate’); Usage: Yes/No; Preference: Open-ended	Four items were constructed by the authors to gauge the availability, utilization, and preference of workplace mental health resources. Participants were asked to: 1) indicate the availability of resources; 2) whether they would use the resources available for them; 3) for those who answer “no” in (2), to provide reasons in an open-ended format; and 4) indicate the preferred type of workplace mental health resource in an open-ended format.	N/A
Work-related constructs				
Relational justice	Adapted from items used in Kivimäki et al. (2003).	1–5 (1 = ‘‘very little’’ to 5 = ‘‘very much’’)	The items assessed whether an individual: (1) considers the respondent’s viewpoint; (2) can suppress personal biases; (3) treats the respondent with kindness and consideration; and (4) takes steps to cooperate with the respondent in a truthful manner. Higher scores indicate higher relational justice in the workplace.	0.84
Effort-reward imbalance	Adapted from the items used in Kivimäki et al. (2007).	1–5 (1 = ‘‘very little’’ to 5 = ‘‘very much’’)	‘Effort’ was asked about with a single question: “How much do you feel you invest in your job in terms of skill and energy?”. ‘Reward’ was assessed with a scale containing three questions about feelings of receiving a return from work in terms of: (1) income and job benefits; (2) recognition and prestige; and (3) personal satisfaction. The scoring method followed Siegrist et al. (2004) [64], in which the ratio between effort and reward was calculated by averaging the scores of the three ‘reward’ items and divided by the ‘effort’ score. Higher values indicate an imbalance between high costs and low rewards.	0.74
Job-demand-control	The Swedish Demand–Control–Support Questionnaire (DCSQ; (Sanne, Torp, Mykletun, & Dahl, 2005)	1–5 (1 = ‘‘very little’’ to 5 = ‘‘very much’’)	Two questions assessed psychological demands by asking whether the worker had sufficient time for the assigned task and any conflicting demands. Another two questions asked about decision latitude, i.e., control, in which the worker can decide on how to conduct the work and what should be done. Finally, a question assessing social ‘support’ asked whether there is good collegiality at work. In order to make sense of these components, an ‘isostrain’ index was formulated by taking job strain (demand divided by control) divided by ‘support’. A higher index score indicated higher job demands in the context of low control and low social support.	0.71
