# Supplementary material for: Socioeconomic Status, Mental Health, and Workplace Determinants among Working Adults in Hong Kong: A Latent Class Analysis
Source: Int J Environ Res Public Health. 2021 Jul 26;18(15):7894. doi: 10.3390/ijerph18157894 (PMC8345757; doi:10.3390/ijerph18157894)
Supplement: Supplementary file 1 [file ijerph-18-07894-s001.zip › ijerph-1249423-SI.pdf]

Online Supplement (Tables)

Table 1.

Measurement Tools

Construct	Scale	Scoring	Description	Reliability
Mental health-related constructs				
Depressive symptoms	The Patient Health Questionnaire-2 (PHQ-2; Kroenke et al., 2003)	0-3 (0 = “not at all”, 1 = “several days”, 2 = “more than half the days”, 3 = “nearly every day”)	The PHQ-2 was developed based on the long form as a tool for preliminary screening of depression. The respondents were asked to rate the frequency of occurrence of two depressive symptoms (anhedonia and depressed mood) over the past two weeks by choosing one of the following four response options: “0-Not at all”, “1-Several days”, “2-More than half the days”, or “3-Nearly every day”. The total scores range from 0 to 6 and higher scores indicate more severe depressive symptomatology. Using a cut-off of 3, the PHQ-2 has a sensitivity of 82.9% and specificity of 90% for diagnosis major depressive disorder.	0.60
Anxiety symptoms	The Generalized Anxiety Disorder 2-item (GAD-2; Kroenke et al., 2007)	0-3 (0 = “not at all”, 1 = “several days”, 2 = “more than half the days”, 3 = “nearly every day”)	The GAD-2 is a simple initial screening tool for generalized anxiety disorder developed based on the long from. It reflects how often the subjects have suffered from first two core symptoms of generalized anxiety disorder (felling nervous, anxious or on the edge & not able to stop or control worrying) over the past two weeks. GAD-7 scores range from 0 to 6 with higher scores representing more severe anxiety symptoms. Using a cut-off of 3, the GAD-2 has a sensitivity of 86% and specificity of 83% for diagnosis generalized anxiety disorder.	0.72

Flourishing	The Flourishing Scale (FS; Diener et al., 2010)	1-7<br>(1 = “strongly disagree”, 7 = “strongly agree”)	The FS consisted of 8 items measuring the respondent's self-perceived success in important areas such as relationships, self-esteem, purpose, and optimism. The scale provides a single psychological well-being score. respondents rated the extent in which they agreed or disagreed the 8 statements relating to their wellbeing, for instance, ‘I lead a purposeful and meaningful life’, ‘My social relationships are supportive and rewarding’ and ‘I am optimistic about my future’. The higher scores represents a person with many psychological resources and strengths and thus more flourished.	0.82
Help-seeking	Self-constructed	Intention:<br>0-10<br>(0 = ‘‘no at all willing’’, 10 = ‘‘most willing’’);<br>Behaviour:<br>0-10<br>(0 = ‘‘did not attend at all’’, 10 = ‘‘most often’’)	Respondents’ intention towards help-seeking was assessed using 3 self-constructed questions asking whether they were willing to 1: seek professional help when facing psychological distress, 2: encourage acquaintances to seek psychological services when needed, 3: discuss mental health issues with others. They would rate their level of willingness. There was an additional question assessing their actual help-seeking behaviours by asking how often do they attend mental health-related activities.	0.73
Workplace mental health resources	Self-constructed	Availability:<br>1-5, (1 = ‘none’ to 5 = ‘adequate’);<br>Usage:<br>Yes/No;<br>Preference:<br>Open-ended	Four items were constructed by the authors to gauge the availability, utilization and preference of workplace mental health resources. Participants were asked to 1) indicate the availability of resources; 2) whether they would use the resources available for them; 3) for those who answer ‘no’ in (2), to provide reasons in an open-ended format; and 4) indicate the preferred type of workplace mental health resource in an open-ended format.	N/A

Work-related constructs				
Relational justice	Adapted from items used in Kivimäki et al. (2003).	1-5<br>(1 = ‘‘very little’’ to 5 = ‘‘very much’’)	The RJ assesses whether an individual 1) considers the respondent’s viewpoint, 2) is able to suppress personal biases, 3) treats the respondent with kindness and consideration and 4) takes steps to deal with you in a truthful manner. Higher scores indicate higher relational justice at workplace.	0.84
Effort-reward imbalance	Adapted from the items used in Kivimäki et al. (2007).	1-5<br>(1 = ‘‘very little’’ to 5 = ‘‘very much’’)	‘Effort’ was asked with a single question: “How much do you feel you invest in your job in terms of skill and energy?’. ‘Reward’ was assessed with a scale containing 3 questions about feelings of getting a return from work in terms of (1) income and job benefits, (2) recognition and prestige, and (3) personal satisfaction. Scoring method followed Siegrist et al. (2004) in which a ratio between effort and reward was calculated by averaging the scores of the three ‘reward’ items and divided by the ‘effort’ score. Higher values indicate imbalance between high costs and low rewards.	0.74
Job-demand-control	The Swedish Demand–Control–Support Questionnaire (DCSQ; (Sanne, Torp, Mykletun, & Dahl, 2005)	1-5<br>(1 = ‘‘very little’’ to 5 = ‘‘very much’’)	there were two questions assessing psychological demands asking about whether the worker has sufficient time for the assigned task and if there were conflicting demands. Another two questions asked about decision latitude, i.e. control, in which the worker can decide on how to carry out the work and what should be done. Finally, a question assessing social support asked whether there is good collegiality at work. To make sense of these components, an ‘Iso-strain’ index was formulated by taking job strain (demand divided by control) divided by support. A higher index score indicates higher job demands in a context of low control and low social support.	0.71

Table 2.

Comparisons of LCA Models with Different Number of Latent Classes

Model	Log-Likelihood	AIC	BIC	SSABIC	Entropy	Class count of the smallest class	LMR LR p-value	ALMR LR p-value	BLRT p-value
1-Class	-7111.81	14263.62	14361.91	14298.39	-	1007	-	-	-
2-Class	-6811.29	13704.57	13906.08	13775.86	0.675	488	0.0019	0.002	<0.0001
**3-Class**	**-6645.91**	**13415.81**	**13720.53**	**13523.61**	**0.801**	**283**	**0.0206**	**0.0211**	**<0.0001**
4-Class	-6539.02	13244.04	13651.96	13388.35	0.816	140	0.839	0.8401	<0.0001

Note. AIC: Akaike information criterion; BIC: Bayesian information criterion; SSABIC: Sample-size adjusted Bayesian information criterion; LMR LR: Lo-Mendell-Rubin likelihood ratio test. ALMR LR: Adjusted Lo-Mendell-Rubin likelihood ratio test; BLRT: Bootstrap likelihood ratio test.

Table 3.

Item-response Probability for a 3-class Model

Variable	Scale/Category	Frontline workers (n=392)	Established leaders (n=332)	Emerging executives (n=283)
Latent class prevalence	-	.38	.34	.28
Gender	Male	**.54**	**.58**	.42
	Female	.47	.42	**.58**
Income (HKD)	$14,999 or below	.30	<.01	.12
	$15,000-$39,999	**.66**	.25	**.83**
	$40,000-$69,999	.03	.46	.05
	$70,000 or above	.01	.28	<.01
Highest education attainment	Below primary	.06	<.001	<.001
	Secondary	**.90**	.11	<.01
	Tertiary	.03	**.89**	**.99**
Age	18-29	.10	.02	**.59**
	30-39	.19	.25	.30
	40-49	.25	.38	.10
	50-59	.32	.29	.02
	>60	.14	.06	<.01
Position	Professional, Managers, Executive	.18	**.82**	.43

	Self-employed / Entrepreneurs	.05	.08	.01
	Office / non-office skilled	.36	.07	.30
	Office / non-office non-skilled	.41	.03	.25
Industry	Commercial Sector	.14	.31	.21
	Semi-professional / Professional	.12	.38	.32
	Hospitality	.15	.03	.04
	Retail & Sales	.17	.07	.09
	Construction / Manufacturing	.20	.14	.11
	Public Services	.05	.06	.09
	Media	.02	<.001	.08
	Logistics / Transport	.14	.02	.06

Note. Item-response probability >.50

Table 4.

General Demographics of Respondents

Variable	Scale/Category	Entire sample (n=1007)	Frontline workers (n=392)	Established leaders (n=332)	Emerging executives (n=283)	Between class differences	Post-hoc tests / pairwise comparisons[1]		
		n(%) / M(SD)	n(%) / M(SD)	n(%) / M(SD)	n(%) / M(SD)	χ^2	FW vs EL Mean Diff/ χ^2	FW vs EE Mean Diff/ χ^2	EL vs EE Mean Diff/ χ^2
SES									
Gender	Male	524 (52%)	210 (53.6%)	200 (60.2%)	114 (40.3%)	24.99***	3.26	11.63**	24.35***
	Female	483 (48%)	182 (46.4%)	132 (39.8%)	169 (59.7%)				
Income (HKD)	$14,999 or below	140 (13.9%)	107 (29.7%)	0 (0%)	33 (12.2%)	616.00***	412.31***	29.92***	324.15***
	$15,000-$39,999	532 (52.8%)	242 (67.2%)	65 (21.6%)	225 (83.3%)				
	$40,000-$69,999	169 (16.8%)	9 (2.5%)	148 (49.2%)	12 (4.4%)				

	$70,000 or above	90 (8.9%)	2 (0.6%)	88 (29.2%)	0 (0%)					
Highest education attainment	Below primary	24 (2.4%)	24 (6.2%)	0 (0%)	0 (0%)	873.71***	594.18***	645.88***	24.05***
	Secondary	386 (38.3%)	359 (92.3%)	27 (8.2%)	0 (0%)					
	Tertiary	590 (58.6%)	6 (1.5%)	303 (91.8%)	281 (100%)					
Age	18-29	206 (20.5%)	36 (9.3%)	3 (0.9%)	167 (59.4%)	485.40***	47.83***	291.41***	341.50***
	30-39	236 (23.4%)	73 (18.9%)	76 (23%)	87 (31%)					
	40-49	250 (24.8%)	96 (24.8%)	131 (39.7%)	23 (8.2%)					
	50-59	229 (22.7%)	127 (32.8%)	99 (30%)	3 (1.1%)					
	>60	77 (7.6%)	55 (14.2%)	21 (6.4%)	1 (0.4%)					
Position	Professional, Managers, Executive	465 (46.2%)	77 (19.9%)	273 (82.7%)	115 (41.1%)	357.61***	338.58***	40.34***	184.58***
	Self-employed / Entrepreneurs	50 (5%)	16 (4.1%)	31 (9.4%)	3 (1.1%)					

	Office / non-office skilled	244 (24.2%)	138 (35.7%)	19 (5.8%)	87 (31.1%)				
	Office / non-office non-skilled	234 (23.2%)	153 (39.5%)	7 (2.1%)	74 (26.4%)				
Industry	Commercial Sector	210 (20.9%)	55 (14.4%)	103 (31.7%)	52 (18.9%)	202.93***	142.45***	93.66***	48.07***
	Semi-professional / Professional	258 (25.6%)	49 (12.8%)	116 (35.7%)	93 (33.8%)				
	Hospitality	77 (7.6%)	56 (14.6%)	10 (3.1%)	11 (4%)				
	Retail & Sales	113 (11.2%)	64 (16.7%)	24 (7.4%)	25 (9.1%)				
	Construction / Manufacturing	151 (15%)	77 (20.1%)	46 (14.2%)	28 (10.2%)				
	Public Services	61 (6.1%)	18 (4.7%)	18 (5.5%)	25 (9.1%)				
	Media	29 (2.9%)	8 (2.1%)	0 (0%)	21 (7.6%)				
	Logistics / Transport	78 (7.7%)	54 (14.1%)	6 (1.8%)	18 (6.5%)				
	Others	6 (0.6%)	2 (0.5%)	2 (0.6%)	2 (0.7%)				

Note. *<.05, **p<.01, ***p<.001; [1]FW: Frontline workers, EL: Established leaders, EE: Emerging Executive

Table 5.

Appraisal of Mental Health Resources at Work

Domain	Category	Entire sample (n=1007) n(%) / M(SD)	Frontline workers (n=392) n(%) / M(SD)	Established leaders (n=332) n(%) / M(SD)	Emerging executives (n=283) n(%) / M(SD)
Evaluation of resources at work					
Sufficiency of mental health resources provided at workplace	Mean scores	1.32 (1.25)	1.26 (1.27)	1.46 (1.28)	1.24 (1.19)
	No service at all	366 (36.3%)	155 (41.6%)	110 (34.4%)	101 (36.2%)
	Insufficient	218 (21.6%)	72 (19.3%)	64 (20.0%)	82 (29.4%)
	Neither sufficient nor insufficient	97 (9.6%)	39 (10.5%)	34 (10.6%)	24 (8.6%)
	Sufficient	291 (28.9%)	107 (28.7%)	112 (35%)	72 (25.8%)
Usage mental health resources provided at workplace	Will use and is currently using	127 (12.6%)	55 (14.3%)	38 (11.7%)	34 (12.1%)
	Will use in the future if needed	728 (72.3%)	274 (71.4%)	238 (73.5%)	216 (77.1%)

	Will not use	133 (13.2%)	55 (14.3%)	48 (14.8%)	30 (10.7%)
Reasons of not using[1]					
	No demand for extra support	81 (60.9%)	42 (76.4%)	31 (64.6%)	8 (26.7%)
	Lack of trust in mental health services	33 (24.8%)	7 (12.7%)	14 (29.2%)	12 (40%)
	Accessibility issue	14 (10.5%)	7 (12.7%)	1 (2.1%)	6 (20%)
	Fear of disclosure	16 (12.0%)	1 (1.8%)	6 (12.5%)	6 (20%)
	Work-related concerns	3 (2.3%)	1 (1.8%)	1 (2.1%)	2 (6.7%)
	Lacking mental health literacy	2 (1.5%)	1 (1.8%)	0 (0%)	1 (3.3%)
	Other	4 (3.01%)	1 (1.8%)	2 (4.2%)	1 (3.3%)
Needs[2]					
No such need	N/A	52 (5%)	34 (8.7%)	13 (3.9%)	5 (1.8%)
Learning resources	Seminars or workshops	413 (41%)	147 (37.5%)	148 (44.6%)	118 (41.7%)
	Online courses	281 (28%)	104 (26.5%)	93 (28.0%)	84 (29.7%)
	Continuing education programme	1 (0.1%)	1 (0.3%)	0 (0%)	0 (0%)
Allowance/financial resources	Medical insurance coverage on mental health conditions	651 (65%)	226 (57.7%)	214 (64.5%)	211 (74.6%)

	Fringe benefits	3 (0.3%)	1 (0%)	1 (0.3%)	1 (0.4%)
	Salary adjustment	2 (0.2%)	1 (0.3%)	1 (0.3%)	0 (0%)
Mental-health friendly policies	Policy catering mental ill-health conditions	635 (63%)	241 (61.5%)	208 (62.7%)	186 (65.7%)
	On-site coach/psychologist	386 (38%)	120 (30.6%)	132 (39.8%)	134 (47.3%)
	Work-life balance policy	2 (0.2%)	0 (0%)	1 (0.3%)	1 (0.4%)

Note. 1: n divided by the number of people who answered 'Will not use'; 2: n divided by the total number of respondents
